# Abstracts and Gleanings

**Published:** 1881-04-20

**Authors:** 


**Treatment of Ozæna.**—The *Lancet*, Jannary 1st, says: In several cases of chronic inflammation of the nasal and pharyngal cavities, giving rise to offensive discharge, Dr. Poore has found decided benefit result from the use of a stimulant and antiseptic snuff having the following formula: biborate of soda, nitrate of bismuth, of each one drachm; disulphate of quinine, ten grains; iodoform, five grains. This snuff has the effect of stopping the fetor and greatly diminishing the amount of discharge from the nostrils. It is liable, as are all snuffs when used for similar conditions, to cake in the nostrils, and it is, therefore, necessary to thoroughly wash out the nostrils once a day. This may be done by means of a nasal dourche, or the patient may easily be taught to snuff a lotion up the nose and allow it to run out of the mouth. A tablespoonful of glycerole of borax dissolved in a wineglass of tepid water forms an exellent wash for the nose, and with a little instruction patients learn how to wash out their nasal and pharyngeal cavities without the aid either of syringe or douche apparatus. In caces where the ozæna is of a simple kind, not due to caries or necrosis of bone, but rather to a sluggish, inflammatory action occurring in a scrofulous subject, considerable benefit is often derived from the administration of the sulphide of calcium in doses of half grain or a grain (in pills), taken three times a day. It is often necessary to cleanse the nasal and pharyngeal cavities with a brush inserted through the interior nares, and also behind the soft palate, so as to reach the summit of the pharynx. The brush may be moistened with glycerole of tannin, and after the cavities have been cleaned a little dry iodoform may be passed into the cavities on the tip of the brush.—*Indiana Medical Reporter.*

### ABSTRACTS AND GLEANINGS.

**Experiments with the Inhalation of Sulphuric Ether.**—Dr Taylor, in the New York *Medical Record*, says: In 1872 I inhaled ether myself for the purpose of experiencing in my own person its physiological effects and noting the amount of rational consciousness remaining while insensibility to pain continued. My experiment resulted in a perfect demonstration to my own mind of the following propositions:

First.—Ether affects the *terminal ramifications* of the nerves *first*.

Second.—That complete insensibility to pain may be produced by ether, while entire rational consciousness remains. Thus, while perfectly conscious of everything, I could stick a pin or make a small incision in any part of my body without feeling any pain whatever. I also noticed that the peculiar deadness or numbness began in the feet and hands first, and extended gradually to the trunk, until—although fully conscious *mentally*—I did not know that I was breathing unless I *looked* at my chest and *saw* it rise and fall. Finally, I was only aware of the cool feeling produced on the tip of my nose by the passage in of the air, and then complete oblivion, lasting, however, only half a minute. I had my watch in my lap and noted the time from my last recollection to my first awakening, for, of course, the handkerchief containing the ether fell from my hand during unconsciousness, and destroyed the continuous effect of the anæsthetic.

Third.—Ether mixed with *much atmospheric air* can be inhaled for hours without producing unconsciousness—sufficient for the quiet performance of a delicate surgical operation. Although sensibility to pain may be abolished during the whole time, yet reflex movements will occur on the application of a sharp stimulus.

Fourth.—That ether almost *undiluted* with air, after the few free respirations, produces anæsthesia and *mental unconsciousness* with complete muscular relaxation, amply sufficient for the uninterrupted performance of any capital operation.

Fifth.—That the condition above-mentioned, being once produced, can be easily maintained for hours by slight additions of ether diluted with air.

Sixth.—That ether *increases* the blood-pressure, and that it enfeebles the pulse only after long-continued and excessive administration. That it gradually produces an increasing slowness and shallowness of respiration, and that in my opinion it kills only in this way, and that if the heart should become enfeebled it is a secondary result of the pulmonary lethargy.

In conclusion, I would say that I have seen great benefit result from the moderate inhalation of ether where cavities existed in the lung; that I have given it to patients afflicted with nearly every form of disease, without having occasion to be dissatisfied with its action as an anæsthetic or alarmed at its operation as a narcotic. I believe that the trouble in producing anæsthesia with ether, complained of by surgeons, is due to the fact that they are afraid to administer it *undi-*

*luted* with air. *I* always use Lente's improved inhaler—an impervious brass nose-piece with rubber stuffing on the edge to make it air-tight. I have always given ether *undiluted*, with the exception of a few inhalations at first to accustom the air-passages to the vapor. Given in this way, I believe it is perfectly safe and efficient. The fearful mortality of chloroform speaks for itself in the report of the eminent men referred to above, and it would be needless for me to discuss the question. Ethidene is only spoken of as *less* dangerous than chloroform; but why use an anæsthetic that is dangerous *at all*, when ether is as safe as anything of the kind can ever be, solely for the purpose of saving a few minutes' time? Chloroform is tricky and uncertain. Ether can only kill, in my opinion, by stopping the respiration, which can be easily watched and the administration controlled to suit any emergency. The few deaths recorded against ether represent so small a percentage as to be almost infinitesimal, while you cannot read a journal without noting one or more deaths from chloroform.

**The Etiology of Vertigo.**—At a meeting of the Medico Chirurgical Society of Edinburgh, in January, Mr. McBride read a paper on the etiology of vertigo, of which the following is an abstract; In relation to this subject the theory was advanced that a nerve impulse striking the brain may, according to its intensity, involve a large or small central area. For instance, the pain caused by a carious tooth might be confined to those fibres of the fifth nerve corresponding to the tooth, or the impulse communicated along these fibrils might overflow and involve the central termination of other fibrils, and even other nerves, producing earache, faceache, and mastodynia. It was, therefore, urged that nerve impulse communicated along any one nerve may overflow and involve other brain area. Shock was considered to be an overflow of nerve impulse so powerful as to involve centres necessary for life. It was argued that vertigo is a definite sensation, and that for any sensation to be experienced a definite encephalic tract must become active. From experiments on animals, the effects of rotation in the human subject, and the phenomena of Meniere's disease, it is known that stimulation of the nerves of the semicircular canals produces vertigo, and that therefore their central terminations must be in close relation with a vertiginous centre, which may (at least physiologically) be assumed to exist. We know, further, that irritation of the canals, if severe, is apt to cause vomiting, syncope, nystagmus, and other phenomena. It is therefore probable that nerve impulse communicated to the vertiginous centre tends to overflow and involve other centres (*e. g.*, the vomiting, cardiac, inhibitory, and oculo-motor centres.) Probably all of these centres are in physiological relation (the central termination of the nerves of the semicircular canals, the vomiting, and the oculo-motor, as also the cardiac inhibitory); therefore impulses communicated to one tend to overflow to the others. In some persons it is justifiable to assume that a nerve impulse tends to overflow more readily on account of unstable equilibrium of the nerve centres, or perhaps of particular nerve centres. Nerve impulse also seems to spread in a definite physiological direction, as seen in the constant results of accidental or experimental lesions of the semicircular canals in man and the lower animals. Possibly this line of argument may be applied to the so-called explosive neuroses. If it be once admitted that vertigo may be produced by impulse applied to the oculo-motor centre, we have here an explanation of those cases where vertiginous phenomena are produced in animals after section of both auditory nerves. Vertigo may originate from causes (1) visceral (2) ocular, (3) aural, as well as central (epilepsy, etc.) and general (anæmia, etc.) causes, as also through nerves of common sensation (pain). The general conclusions arrived at were—

1. That there is a cerebral area or tract, stimulation of which produces vertigo.

2. That this area may be stimulated by impressions ocular, auditory, sensory, or visceral, as well as central changes, and is in intimate physiological relation with the vomiting and oculo-motor centres. That is to say, impressions conveyed to one of those centres tend to involve the others by overflow of nerve energy.

3. The excessive stimulation of the vertiginous area will produce an overflow of nerve impulse to various motor centres, and probably unconsciousness.

4. That inasmuch as the phenomena of its excessive stimulation (as in rotated men or animals, Meniere's disease, etc.,) are represented by a different train of symptoms, we may infer that overflow of nerve impulse usually proceeds in the same direction—in other words, involves the same centres—first those which are intimately connected with it, then those more remote.

5. That possibly the same process of reasoning may be applied to death from shock, epileptic attacks, convulsions, and some of the other so-called explosive neuroses.

6. That in typical cases, ocular, stomach, and auditory vertigo may be distinguished from one another by the centre first involved. Thus we should expect that stomach vertigo would be preceded by nausea, if not vomiting; that ocular vertigo would be caused by changes in the motor apparatus of the eyeball, but that in auditory vertigo the giddiness is the first and may be the only symptom.

7. The sea-sickness is probably generally due to stomach irritation, for here, as a rule, we have first vomiting, and afterwards vertigo, which is, however, by no means a constant accompaniment.—*Medical and Surgical Reporter.*

**Treatment of Indigestion and Heartburn.**—In the course of an article in the *Practitioner*, January, 1881, Dr. J. Milner Fothergill writes:

For the purpose of whetting the appetite and thus acting reflexly upon the gastric secretion, we employ the class of agents known as bitters. To these we add hydrochloric acid. Ringer has pointed out how an alkali taken into the stomach before a meal, when the stomach is alkaline, produces a freer flow of acid afterwards. Consequently we comprehend the value of that well known preparation indifferently termed, "Haust. Stomach," or "Mist. Mirabilis," or "Mist. Rhei et Gentian," in the various hospitals; a combination of world-wide fame. One drawback to this combination of rhubarb, gentian and soda is, that the student becomes familiar with it and its virtues, but remains ignorant of its exact composition, and so loses sight of it when he enters upon practice for himself. Such a mixture before meals, followed by ten drops of hydrochloric acid after the meal, will often make the difference betwixt imperfect digestion, producing discomfort, and digestion so perfect that it does not provoke consciousness. Or where there is much irritability in the stomach, *i. e.* when a bare, red tongue imperfectly covered with epithelium suggests a like condition of the internal coat of the stomach, then bismuth is most soothing. The mixture of soda, bismuth, and calcimba is in use for such indigestion with good results. The dietary in such a case should consist of the blandest food, milk, with or without baked flour in it, beef tea with baked flour; nothing more till an improved condition of the tongue tells of a more normal condition of the stomach. In such cases a plain opium pill at bedtime often soothes the stomach very nicely. Then there are cases where imperfect digestion is accompanied by the production of fatty acids, butyric and others, which add the phenomenon of "heartburn" to the symptoms; or there may be later products formed which cause the bitter, hot taste in the mouth on awakening in the morning or after a post-prandial nap. It is usual to treat "heartburn" by the exhibition of an alkali; but this is not good practice. In union with an alkali the offending matter is nearly as objectionrble as in the form of free acid. It is much better to give a mineral aeid, as the hydrochloric, or phosphoric, which breaks up the feebler organic acid. By such means we can aid the digestive act. Then at other times the indigestion is due to lithiases, where the presence of uric acid impairs the efficiency of the gastric juice. In these cases all measures which do not entertain the causal relations of dyspepsia are of little use. By the administration of potash in a bitter infusion, well diluted, taken half an hour before a meal, this element of trouble is removed. In all cases of gouty persons suffering from dyspepsia, do not forget this cause of impairment of the gastric juice.—*Medical and Suvgical Reporter.*

**The Tongue as a Diagnostic Point in Diseases.**—From an extended observation I have deduced several important deductions from the appearance of the tongue which have been specially valuable as a diagnostic point in various phases of disease.

The (saburra) or fur of the tongue should be specially observed.

FIRST.—A heavy coated tongue, especially at its base, with a deep yellow coat, the liver is the cause, still tobacco chewers may have that and the liver be sound. I have noticed in several instances jaundice with a white coated tongue.

SECOND.—The pinched and shrunken tongue shows atony of the digestive tract as found in dyspeptic troubles.

The elongated tongue shows irritated condition with determination of blood to the stomach and intestines.

It will also exist in excitation of the nervous system.

It is specially found in children. In these conditions irritant cathartics *do* harm.

THIRD.—The dry tongue has several very important considerations as indicating in febrile conditions, much danger which demands close attention. Food must be taken in such cases in fluid form, bearing in mind that it always must be above the temperature of 100°, of a nutritious character, which may easily be assimilated. Dryness may be found in vascular excitement, more particularly associated in excitation of the ganglionic and nervous system. Hence the indications for sedatives for the vascular excitement, and diaphoretics for contraction and excitement of the nervous system, together with other proper medication. When the tongue changes from the dryness to a brown or black color, with sordes about the teeth, it means that the blood is in a specific condition—and not, as commonly supposed, due to a typhoid condition. Hence, a marked improvement always follows from the best antiseptics with stimulants and tonics.—*Dr. Fox, in Indiana Medical Reporter.*

**A Case of Phlyctenular Conjunctivitis.**—Dr. W. W. Seely said: The case I now present represents a class of frequent applicants at the dispensary.

You can usually tell them at a glance, for the child comes in with its eyes closed and head down, shunning the light as much as possible. When I lay its head between the knees, I find one or two well-marked phlyctenulæ quite near the sclero corneal junction. These phlyctenulæ are (usually) small vesicles, in the beginning, filled with fluid containing many little cells. Shortly the epithelium covering the vesicle becomes shed, and we have a veritable little ulcer, exposing branches of the sensitive nerve; and this irritation extends to the nerves supplying the orbicular muscle of the lids, producing the blepharo spasm.

Very often, as an accompanying phenomenon, we have more or less catarrhal conjunctivitis. These phlyctenulæ may be quite numerous or occur singly, be situated some distance from the cornea, just on the limbus, or rest, like a saddle, partly on the conjunctiva partly on the cornea, with a marked tendency to extend upon the latter.

For phlyctenulæ, not involving the cornea to any extent, the local treatment usually suffices for relief, and is extremely simple.

It is never necessary to use, what is so often recommended, viz: atropia; since you will find that it often adds to the irritability of the eye.

We formerly were in the habit of waiting till the irritation had subsided somewhat before beginning the local treatment, simply because the remedies used were in themselves irritating.

This case presents vesicles not yet ruptured, though you see I apply yellow oxide in vaseline (5 grains to the half ounce) and assure you that to-morrow when the child is brought here it will present a vastly different aspect.

You will find that this remedy can be used right from the beginning, and is altogether the best one and the only one needed so long as the cornea is not involved. If there were many sores about the face, nose or mouth, we would simply use an ointment of vaseline and boracic acid, as you have often seen, it being a perfectly bland application and thoroughly efficient.

I will say in regard to the yellow oxide in these cases, that it has always been used in too strong preparations and in a vehicle that of itself is irritating. The strength I recommend is quite enough. If you are inclined to use an eye-water, let it by all means be eserine instead of atropine, and procure the salicylate of eserine if possible, preparing a little at a time, so that it shall be fresh, *i. e.*, free from sources of irritation.

Your constitutional remedies, as I have said, must depend upon the general state of the child, *i. e.*, whether it is in such a condition as to need internal medication on general principles, when of course your cod liver oil, quinine, and iodide of iron can be given.—*Cincinnati Clinic.*

**Almost a Specific for Catarrh of the Nasal Passage.**—Dr. H. A. Eberle, of Webster City, Iowa, in American Medical Journal, says: In this State, where catarrh is so prevalent amongst the people, any remedy that would perform a cure would be hailed with the greatest delight. Iodoform, as a remedy for chronic ulcers, was used quite extensively in the Montreal general hospital in 1872 to 1876, with such good results as to warrant its trial in private practice. Since that time I have made use of it in very many ways, in the treatment of piles, fissures, granular ulceration of the nterus, etc., and always with gratifying results.

In view of its healing properties, I was led to adopt the remedy for the treatment of catarrh, and as I had been a sufferer from the disease in a chronic form for many years, and had used many preparations with varying success and little benefit, I concluded to make use of it in the following manner: First, an ointment is made thus:

| | | |
|---|---|---|
| ℞ | Iodoform, finely powdered........................................ | grs. lx. |
| | Ext. geranium, solid.................................................. | grs. x. |
| | Acid carbolic............................................................ | gtt. xv. |
| | Vaseline..................................................................... | qs. ℥ i |
| Mix. | | |

Secondly, bougies are made of absorbent cotton saturated with the above ointment and simply introduced up the nasal passages as far as necessary at bedtime. These are left in all night and are easily removed in the morning by blowing the nose.

This is repeated for a week or ten days, when the most obstinate case of catarrh will yield to the treatment. Scarcely any other treatment is necessary, except the occasional use of the posterior nasal douche with some cleansing fluid. I usually employ a weak, tepid solution of chloride of sodium before introducing the "iodoformized cotton" tent.

**Sulphate of Hyoscyamin as a Mydriatic.**—Dr. Risley, in the Philadelphia Medical Times, gives the results of experiments made with the sulphate of hyoscyamin as a mydriatic. From his report of eight cases in which a solution of gr. ii. to the ounce was used, we learn that the above is a powerful mydriatic, which in its rapidity of action over the iris and ciliary muscle and in the duration of its control is more like duboisin than like atropin. Accommodation usually returns in about 100 hours; when used in the given strength there is little danger of annoyance from constitutional impression, only one case out of eight, a lady, having complained of vertigo, which quite disappeared in an hour and a half.—*Monthly Review.*

**Iodine Treatment of Diphtheria.**—Dr. H. P. Gauther communicates the following cases of diphtheria treated with iodine in the practice of Prof. Jay Owens, of St. Paul, Minnesota:

A woman was taken with sore throat, chilly sensations, difficulty in swallowing, and fever, together with patches on the left tonsil. At 5 p. m. the same day ten-drop doses of Lugol's solution of iodine were ordered every hour. The patient's pulse at this time was 127 and had a temperature of 104½° F. At 1 p. m. the following day she could swallow without difficulty, the patches had almost disappeared from her tonsils, her pulse was 106 and her temperature 101½° F. She was up and about in two days more.

Case II. Male, aged twenty-six, had at 5 p. m., when seen, a pulse of 133 and a temperature of 103¾° F. There was a diphtheritic patch on each tonsil, and the patient was extremely weak. Ten-drop doses of Lugol's solution were ordered every hour in water. At 1 p. m. the day following the patch on one tonsil had disappeared, the other was disappearing; the patient had a pulse of 79, and a temperature of 100° F. In three days he was able to engage in his usual avocations. While it is possible that elements of error may exist in cases observed by a single physician, these can scarcely be expected to occur in the results of two observers, so that from these cases of Dr. Owens, added to those of Dr. Gauther, it would appear that iodine exercises considerable influence over diphtheria, but the cases are still too few to express a decided opinion on.—*Chicago Medical Review.*

**Alstonia Constricta.**—The bark of the Alstonia constricta, or Australian fever-tree, has been known as a pharmaceutical curiosity—at least, since 1863, when Palm separated from it a bitter principle which he called alstonin.

Dr. Bixby, in American Medical Journal, has used the drug largely during eighteen months, and has prescribed it in thousands of cases. He finds that its action resembles in many respects the combined action of quinine and nux vomica. It is an antiperiodic of the highest type, better, in his opinion, than quinine or cinchonidine. It is a cerebro-spinal stimulant and tonic, acts positively upon the great sympathetic nerve-centres, and consequently increases, positively and permanently, the vital forces of the entire system. A proper sedative should be given before the use of the bark is begun.

In general nervous depression it acts like a charm; in typhoid, puerperal and other fevers, in recent colds and rheumatism, it has produced good results.

**Trichina Spiralis.**—Foreign nations have gotten up a big scare on this subject. Importation of this article from the United States is now forbidden by France, Russia, Germany, Italy, Spain, Greece, and Portugal. The consequent injury to American trade from these restrictions is, of course, very great. We export annually about eighty million pounds of pork to France. The amount sent to England is twelve times as great, and the total value of all the meat thus sent out from this country is estimated to be about one hundred million dollars annually.—*N. Y. Med. Rec.*

**Potassium Bromide in Infantile Diarrhœas.** — Dr. F. Charles Lawrence, of Texas, says, in Cincinnati Clinic: During the summer months of 1879, while residing in one of the towns of the Rock River Valley in Illinois, I was daily called to prescribe for children suffering from the various forms of diarrhœa. Many of these cases were the diarrhœa of dentition, in which a predominating morbid element was hyperæsthesia of the nervous system. I gave the bromide in doses of ½ to 2 grains, according to the age of the patient, and generally with intervals of two hours between doses. In 20 cases of this kind, diarrhœa and vomiting ceased after the administration of a few doses, the little patients falling into a quiet sleep. In seven of these cases, I found considerable swelling of the gums, which I ordered rubbed several times daily with potassium bromide, gr. xxx, to glycerine and water aa ʒij. This comprised the entire treatment of the cases, and the results were to me very gratifying.

In another group of cases, children from 3 to 7 years of age, depending upon acidity of *primæ viæ,* with stools more or less mingled with blood, and vomiting often present, the bromide in 1½ to 3 grain doses, with proper hygienic measures, gave good results, only about one case out of seven requiring any astringents or opiates.

**Ovariotomy.**—Ovariotomy, first successfully performed in this country, and subsequently styled by Piorry "une audace Americaine," has remained pre-eminently an Anglo-American procedure. The names which stand foremost in the list of surgeons who, since McDowell, have helped to establish the operation, are those of Spencer Wells, Clay, Baker Brown, Keith, Thornton, in Great Britain; of Atlee, Peaslee, Kimball, in the United States; of Pean and Koeberle, on the continent of Europe. But to no one more than to Spencer Wells belongs the honor of having raised ovariotomy, and with it abdominal surgery, to its present secure position, and of having taught the surgical world the best methods for its successful performance.

A few figures will suffice to show the great degree of success now attainable by means of this operation, in experienced hands. Spencer Wells, only a few weeks ago, communicated to the Royal Medical and Chirurgical Society of London, a paper which summarized the results of his last 200 cases, completing a series of 1000 ovariotomies performed by him from first to last. Among the 1000 patients 231 had died and 769 had recovered, the mortality, however, having steadily diminished from 34 in the first 100 to 11 in the last. The last 112 cases took place in private practice, and were all done antiseptically, the result being a mortality of 10-6 per cent.—*Boston Med. Jour.*

**Artificial Vaccine.**—Mr. J. Lawrence Hamilton, of London, proposes to supply an abundant supply of artificial vaccine lymph, produced outside the body of living man or living animal, by isolating and then breeding the vaccine organisms in suitable germ nutritive solutions which have been previously deprived of all septic and other noxious germs. The special precautions which Mr. Hamilton considers necessary to secure success in breeding the artificial vaccine lymph, as well as the results therewith inoculating men and animals, will be published at a future date.—*Boston Med. Jour.*

**On the Abortive and Curative Treatment of Small-Pox.**—"On the 16th of January, 1880," says Dr. Bouyer, of St. Pierre de Fursac, in the *Bull. de Therapeutique*, December 25th, "I addressed to the Academy of Medicine the following letter: 'I have the honor to forward to the Academy a sealed packet containing a formula for what I believe to be the curative and abortive treatment of small-pox, and which I have found very successful in six cases. When I have tried the treatment in a greater number of cases, I shall lay before the Academy the results of my investigation.'"

On the 16th of March, Dr. Bouyer had had fifteen cases, all confirming the efficacy of the remedy, and shortly afterwards he was pleased to make it known. The following is the formula recommended—

| | | | |
|---|---|---|---|
| ℞ | Alcohol ............................ | 10-15 grams (2½–4 ʒ) | |
| | Salicylic acid,...................... | 1 gram (15 grs.) | |
| | Simple syrup,...................... | 20 grams (5 ʒ.) | |
| | Water,.................................. | 50 grams (2 ℥.) | M. |

Of this a tablespoonful should be taken every six hours, if the case is seen early, and every four hours if the disease is well advanced before the treatment is begun. Dr. Bouyer finds that under this treatment commenced early, the eruption is discrete, or if confluent, the pustules are of small size, and contain little pus. They contract between the sixth and eighth day, leaving light furfuraceous crusts, which fall off in a few days without leaving either cicatrices or stigmata. The fever of suppuration is always greatly diminished.—*Med. and Surg. Reporter.*

**The Treatment of Nævus.**—Dr. Richard Bligh says, (in *British Medical Journal*): I am perfectly certain that the ligature is very rarely necessary, and that a very old remedy, the liquor plumbi subacetatis, is far more to be relied on than anything else for destroying nævi of various kinds. I have always found that, after it has been applied regularly for about four months, once a day (if used oftener it will give rise to ulceration), the nævus becomes dotted over with white spots, which gradually coalesce till the nævus disappears. This it will do, without fail, in the course of one or even two years according to size. I had an illustrative case about three years since. A child about four years old had a nævus on the temple about the size of a two-shilling piece, with two or three smaller ones adjoining. It was daily becoming larger and more prominent. The liquor plumbi soon stopped its growth; in a few months, there was a very visible improvement, and for some time now all traces of it have disappeared.—*Indiana Medical Reporter.*

**Antiseptic Treatment of Carbuncle.**—Dr. Schueller lays open the carbuncle in the ordinary manner by a crucial incision, and then, by means of the sharp spoon, thoroughly scrapes away the diseased connective tissue underneath the flaps. After complete disinfection, an antiseptic dressing is applied and drainage established. Recovery usually follows in a few days.—*Inter. Jour. of Med. and Surgery.*

**Tripolith a Substitute for Plaster of Paris.**—Prof. von Langenbeck (*Medical Times and Gazette*) describes a new material for fixative dressings. Its name suggests its properties of hardness and resistance. It was discovered by Mr. B. von Schenke, of Heidelberg. Originally, it was intended for stucco and decorative purposes, for which it was said to be superior to plaster of Paris. While its mode of manufacture is not known, its chief constituents are calcium and silica, with a little of iron oxide. Tripolith bandages are employed in the same manner as those of plaster of Paris. Its advantages are: (1) Tripolith appears to absorb moisture from the atmosphere less freely than plaster, and its power of setting is not lost even after long exposure to the atmosphere. (2) Tripolith bandages are lighter, and therefore pleasanter to the patient—about 14 per cent. lighter. (3) Tripolith dressings harden more quickly than plaster. While a bandage made with the best plaster requires ten to fifteen minutes before it is quite set—and in wet weather remains soft for hours—tripolith sets completely in three to five minutes. On the other hand it gives off vapor for many hours, and even after twenty-four feels moist to the touch. (4) Once hard and dry, tripolith absorbs no more moisture. A piece of dried tripolith dressing undergoes no change when laid in water. It would be possible, therefore, to allow a patient to bathe in his tripolith dressing, provided means were taken to prevent the water getting up inside it by means of an India rubber covering; while as regards plaster, it is necessary to paint it with damar varnish in order to make it water-proof. (5) Tripolith is a trifle cheaper than plaster of paris.—*Detroit Lancet.*

**A New Narcotic—Pitchoury Bidgery.**—Pharmacologists are becoming interested in a new narcotic which has hitherto been known only to the natives of Queensland, by whom it is termed "Pitchoury Bidgery." The plant belongs to the natural order of Solanaceæ, and is indigenous to South Australia. It grows to the height of three or four inches, and bears wax colored flowers with roseate, bell-shaped spots. In the month of August, while the plant is in bloom, the leaves are gathered, dried with steam heat and sent in sacks to market. They are then pressed, similar to plug chewing tobacco, and on being chewed produce a feeling of complete indifference to physical pain or fatigue. In small doses they possess stimulative action; when used moderately they allay hunger and thirst, so that like coca leaves they lessen tissue waste and may be used as an aid to enable persons in enduring excessive exposure or fatigue, there being but a small or limited amount of food taken.—*Monthly Review of Pharmacy.*

**Despotism in Lunatic Asylums** is the title of an article by Mr. Dorman B. Eaton, in the *North American Review* for March. He characterizes the American system of asylum management as follows: "A deceptive and vicious system, adroitly and ably administered, has lulled and misled public opinion; screening abuses by secrecy, shutting out light by arbitrary methods, defying exposure and change by the exercise of a despotic authority which ought never to have been conferred upon the managers of asylums." He suggests remedies for these abuses, drawn from a study of asylum management in Europe.—*N. Y. Med. Rec.*

**Diphtheria.**—Dr. Knox (in *Peoria Medical Monthly,*) says: Clinical experience proves that diphtheria begins either as a *local* or *general* disease. As a local disease, it originates in the fauces and disappears without systemic contamination, or if blood poisoning occur, it is from the local affection.

As a general disease, blood contamination exists before the formation of the pseudo-membrane, and before much faucial inflammation occurs.

Diphtheria also occurs as a secondary affection, especially engrafting itself upon scarlet fever and measles.

The blood poisoning leaves an anæmia from which recovery is slow. Not only is there deficiency of the red corpuscle, but the vital current seems vitiated. Another sequela is paralysis, which usually occurs fourteen or eighteen days after the subsidence of the disease. The most frequent is paralysis of the muscles of the pharynx, next of the extremities; last of the trunk, muscles of respiration and heart. The former generally disappears in from one to four months, the latter are fatal.

**The Organisms in Abdominal Typhus.**—Eberth examined the affected intestines, mesenteric glands, liver and other organs, in twenty-three cases of ileo typhus for micro-organisms, but specially preparations in alcohol. The sections were treated with acetic acid. In twelve cases, between the eleventh and thirteenth day of sickness, numerous conglomerated heaps of bacillii weie discovered. The isolated staffs were four times longer than broad, about one-half as long as the diameter of the red blood-corpuscles, they were rounded threads and the contours were pale. In the second and third week they were most numerous. They are distinguished from the bacillii (from decomposition) occurring in the typhus ulcers, by their more delicate contours, and by their assuming a more intense color when treated with methyl violet and Bismarck brown.

It is very probable, therefore, that these staffs may be considered as typical typhus bacillii.

In eleven cases, between the thirteenth and fifty-sixth day of sickness, these staffs were not found.—C. J. Eberth, Zerich, Virchow's Archiv. Bd. lxxxi, p. 58.—*Clinical Record.*

**Ergot.**—Dr. Keating—*Med. Record*—"records a case wherein a patient was poisoned by half an ounce of fluid extract of ergot, in divided doses. He says: Ergot is a powerful and dangerous drug."

At a recent meeting of the Academie des Sciences, M. Boirsarie read an article on the inconvenience and dangers of ergotin, "when given in *continuous doses* for a considerable period, *even when these are small*, ergotine accumulates in the economy, and may suddenly give rise to ergotism. He gave, also, the history of a case of *spontaneous gangrene* of the *lungs from ergotism.*"—*Phil. Med. Times aud Therap. Gazette.*

Dr. Wm. Commons—"Ergot, its uses and abuses," says: When given in larger doses Ergot may cause death by spaemodic contraction of the heart, from its specific action upon muscular structure."—*Indiana Medieal Reporter.*

**Hemorrhoids.**—Dr. Blackwood (Trans. Amer. Med. Ass.) says: During the last twenty years my experience has been large, and my deductions are based entirely upon practical results, not upon theory. I am convinced that the subject does not receive the attention it deserves, and with a desire to attract more attention to it, and to summarize in closing, the following points are suggested :—

1. Hemorrhoids may be arrested by proper attention to diet, and to a normal daily evacuation of the bowel.

2. Hemorrhoids, being present, may be generally kept in check, frequently greatly relieved, and sometimes cured entirely, by the means used to prevent them.

3. Hemorrhoids becomming troublesome, despite medical treatment, should be removed surgically without delay.

4. Hemorrhoids may be quickly, surely, and safely removed by tne preferable operation of injection by carbolic acid.

**A Powerful Antiseptic.** — The president of Pharmaceutical Society, of Liverpool, exhibited last week a sample of eugenol, which had been placed in his hands on the previous day. He said it had recently been noticed as a very energetic antiseptic, and it was also said to be a remedy for toothache. Both these properties could be readily understood, as oil of cloves, from which it was obtained, as well as oil of peppermint, had long been used to prevent ink, starch, paste, etc., from becoming mouldy. The oils had also been long regarded as remedies for toothache. It was also known as eugenic or carpopyllic acid, having a formula $C_{10}$ $H_{12}$ $O_2$ and forming salts with bases. He said, it would be interesting to determine its rotatory power as compared with oil of cloves, which he would do before the next meeting.—*British Medical Journal.*

**Notes on the Treatment of Diphtheria**.—In 150 patients suffering with diphtheria, v. C. has lost no case since he has used liq. ferri sesquichlor. locally and internally. Mixed with equal quantities of water, it is painted (a pencil of lint) over the bleeding mucous membrane subsequent to the removal of the pseudo-membranes. Injections of a weaker solution, 1 : 3 aq., are made into the nostrils; internally, the proportion of 10 to 20 drops in 200 of water; a teaspoonful every fifteen minutes. This treatment is continued until no membranes show themselves. It seems as if the most of C.'s patients had passed the age of childhood (42 cases from the school of cavalry in St. Petersburg). He has treated no children under two years of age.—A. v. Collan, St. Petersburger *Med. Wochenschrift,* 1880.—*Clinical Record.*

**Delirium in Acute Rheumatism from Salicylate of Soda.**—In four cases recently reported in British Medical Journal of January 29, salicylate of soda was administered in twenty-grain doses every three hours, with the apparent result of producing delirium. The delirium was of depressing type, and the idea of persecution entered into it in most of the cases. The delirium promptly disappeared on the salicylate being stopped.—*Ibid.*

**A New Method of Inhalation.**—Dr. Feldbausch, Straussburg, (*Int. Jour. of Med. and Surg.* Vol. I, No. 2; *Berlin Klin, Wochenschr.*, No. 47, 1880,) has invented a very convenient apparatus for giving permanent inhalation of volatile substances in treating the various affections of the respiratory tract, as well as for a prophylactic against infectious diseases, etc. It consists of a pair of capsules made to fit the anterior nares, and is nearly or quite invisible when introduced. It contains a piece of flannel or blotting paper on the inner surface of each tube, which is saturated with the therapeutic agent. The carbolic inhalation is the doctor's favorite, which he recommends especially in phthisis, (not only as a disinfectant, but to relieve cough,) and in acute catarrhs.—*Detroit Lancet.*

**To Terminate the Chloroform Narcosis.**—A peculiar device is mentioned by Schirmer in the February number of the *Centralblatt f. Augendeilkunde.* He claims to have used it in his clinic for many years, and often succeeded in producing inspiratory movements when other means failed. He also employed it to induce rapid recovery, for instance in strabismus operations, in order to test the result. The method consists in irritating the nasal mucous membrane. It has long been known, at least to physiologists, that the fifth nerve retains its sensibility longer than any other part in narcosis, and that reflexes may be induced through this nerve when other irritations fail. Schirmer uses simply a rolled piece of paper, which he turns in the nose. In dangerous cases he dips the paper into ammonia.—*Chicago Medical Review.*

**The Treatment of Asthma.**—I wish to draw attention to a method of treatment of asthma, described by Dr. R. B. Faulkner, in the New York *Medical Record* for September 25th. His plan is to paint a strip of iodine over the course of the pneumogastric nerves in the neck. He gives three cases of pure spasmodic asthma, which were relieved of their attacks by this means, after having resisted every other remedy of which he could think.—*Boston Medical Journal.*

**Resection of Rectum.**—It is said that Koeberle has recently resected the rectum in four cases of rectal cancer, resulting in recovery without relapse, and in another case where the intestine was strangulated he resected two yards of the intestines and united the edges of the intestine left with a ligature. The operation was followed by recovery in a month.

THE last use of phosphorus is to prevent infant deformities. A lady in England, who had given birth to three children in succession with club feet and other deformities, took phosphorus during her fourth and subsequent gestations, and bore well-formed children.—*Pac. Med. and Surg. Journal.*

THE question has been raised in England, whether when a working man is isolated because of contagious disease in his family, compensation from public funds should be allowed him for the loss of his labor. It is argued that the subject should be referred to a sanitary authority, with power to grant relief in such cases.

